# Supplementary material for: Predicting the Real‐Valued Inter‐Residue Distances for Proteins
Source: Adv Sci (Weinh). 2020 Aug 10;7(19):2001314. doi: 10.1002/advs.202001314 (PMC7539185; doi:10.1002/advs.202001314)
Supplement: Supplementary file 1 — Supporting Information [file ADVS-7-2001314-s001.pdf]

© 2020 Wiley-VCH GmbH

ADVANCED SCIENCE Open Access

Supporting Information

for *Adv. Sci.*, DOI: 10.1002/advs.202001314

Predicting the Real-Valued Inter-Residue Distances for Proteins

*Wenze Ding and Haipeng Gong**

Copyright WILEY-VCH Verlag GmbH & Co. KGaA, 69469 Weinheim, Germany, 2020.

Supporting Information

Predicting the real-valued inter-residue distances for proteins

*Wenze Ding and Haipeng Gong**

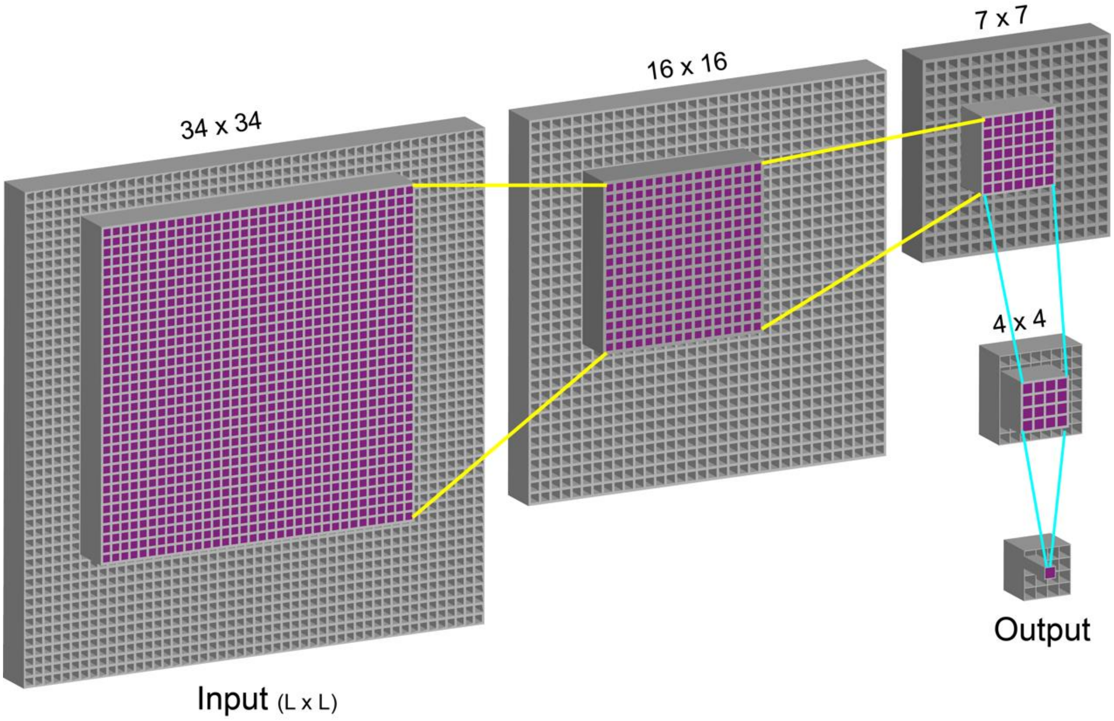

Figure S1. Illustration of the patch classifiers (34×34) in D. The size of patch (purple and highlighted as convex) evolves along sequential convolutional layers, with the size of feature map (gray) changing accordingly. Yellow and blue lines represent convolutional strides of 2 and 1, respectively. Numbers on top of the feature maps list the patch size in each layer.

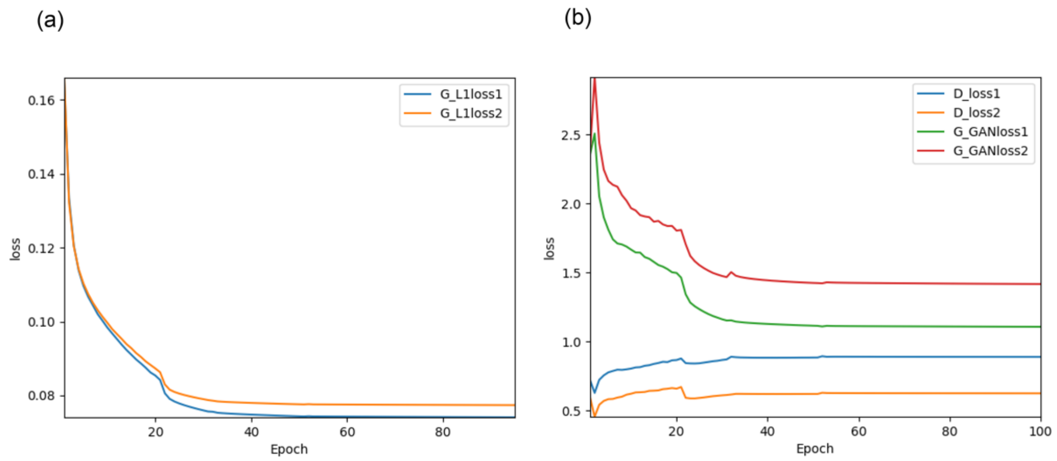

Figure S2. Patch classifiers in D help to improve the model training. The patch classifier of D (implemented as FCN) is marked by the suffix of "1" and the original single classifier (implemented as SPP) that makes judgement on the entire input is marked by the suffix of "2". **(a)** L1 loss of G. **(b)** Loss of D and GAN loss of G. Generally, for a GAN system, the smaller GAN loss of G and larger loss of D both mean that G is more likely to produce reality-indistinguishable fakes to fool D. Thus, the simultaneous reduction of GAN loss of G and enhancement of D loss in the case "1" imply that the distance map produced by G becomes more realistic when the patch classifier is applied.

(a) (b)

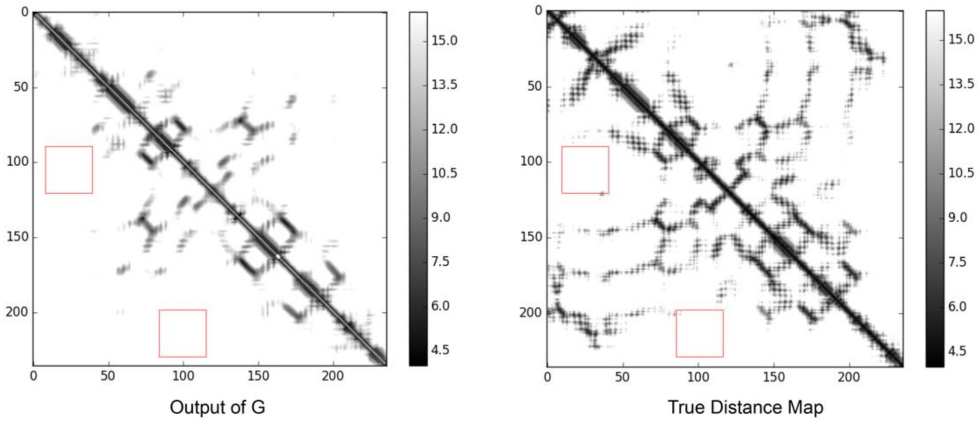

Figure S3. Patches of the blank regions that lack contributive information for folding may cause confusion in the judgement of D. The premature prediction produced by G in the early stage of GAN training (**a**) and the true distance map (**b**) are shown as an example here. 34×34 patches marked by red squares are likely to be identified as true even if many stripes with intense signals are completely missed in (**a**).

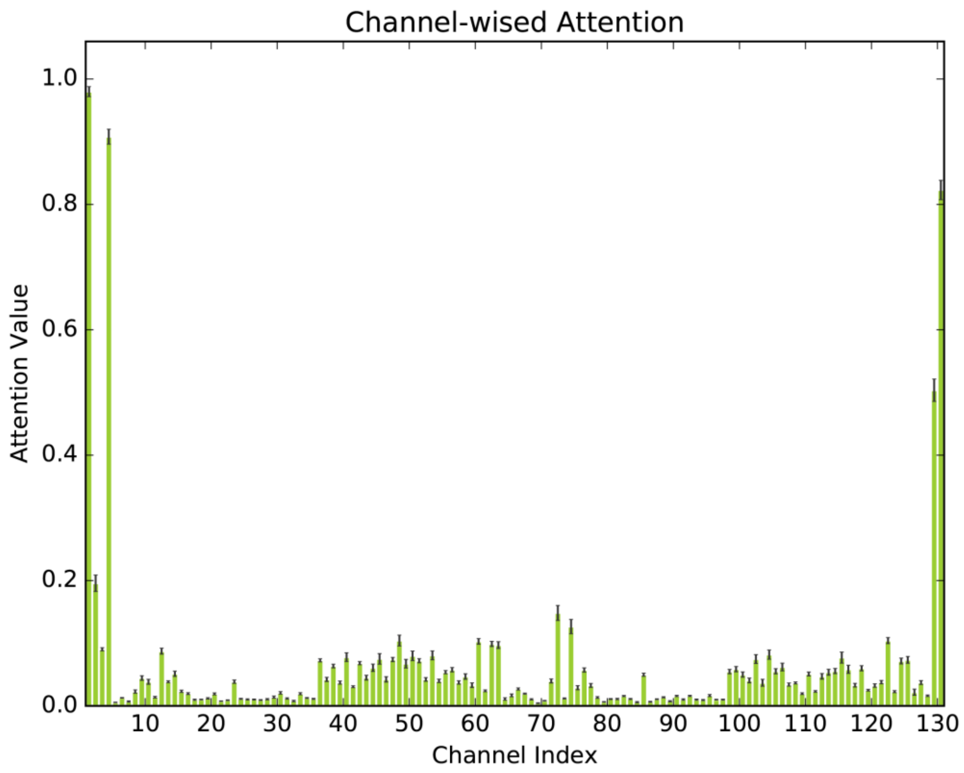

Figure S4. Channel-wised attention. Error bars are added on the head of each pillar. Our input feature has 130 channels in total (see Experimental Section), in which the first four (indices 1-4, corresponding to CCMpred, MI, MSA gap frequency and relative residue position) are 2D features, the latter two (indices 129 and 130, corresponding to target length and MSA count) are 0D features and the rest are broadcast 1D features. Signal strength of 1D features is inhibited dramatically by the channel-wised attention operation.

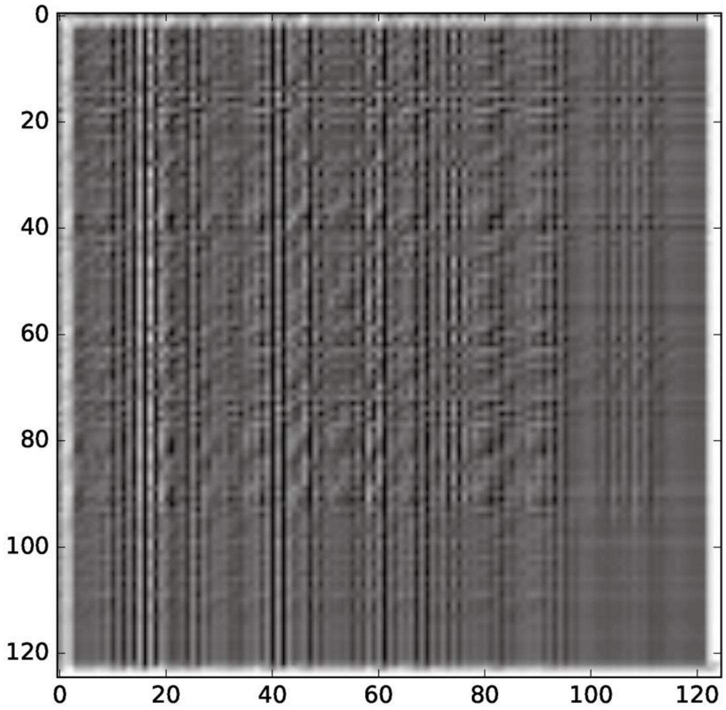

Figure S5. An example of the pixel-wised attention map. Clearly, the weight of each pixel (shown in the grayscale) has been readjusted after the pixel-wised attention operation.

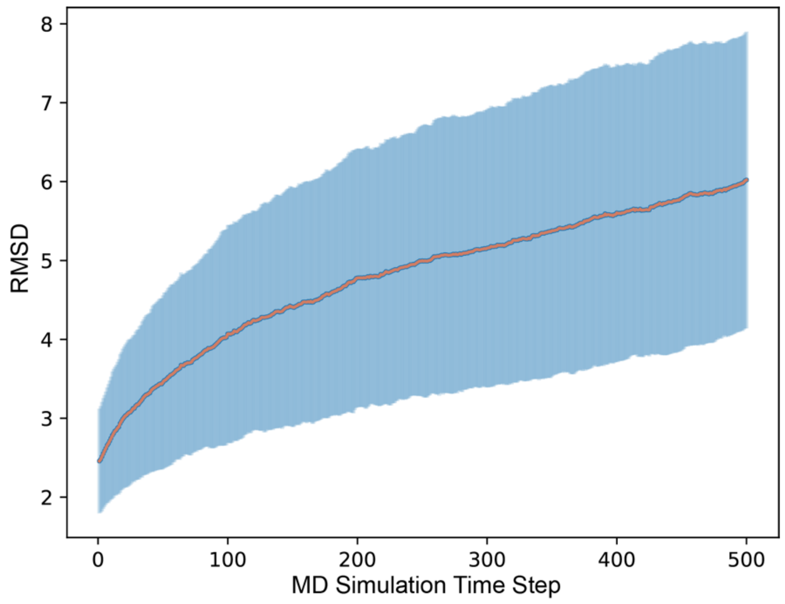

Figure S6. Structure oscillation in MD simulations. The orange line represents the average RMSD of all proteins in our training set along the simulation time step. The blue shadow represents corresponding error bar at each time step.

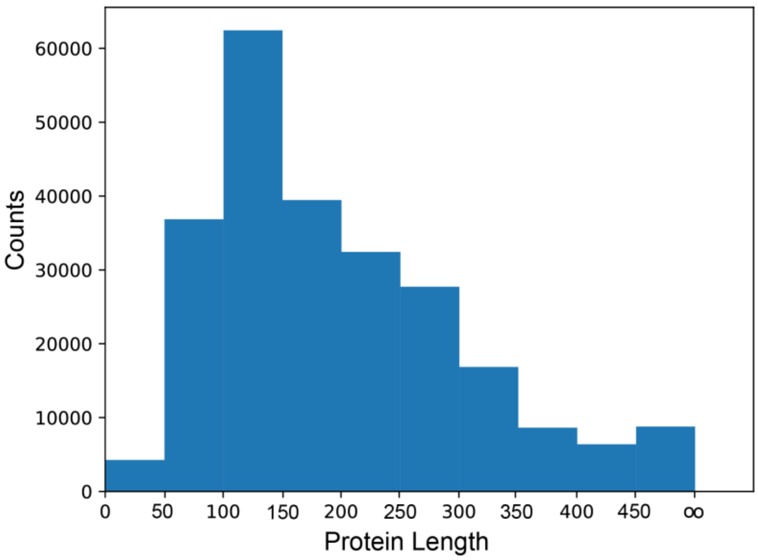

Figure S7. According to the statistical analysis, 203159 out of 243801 (or 83.3%) of the protein domains in the SCOPe (version 2.07) database have no more than 300 amino acid residues.

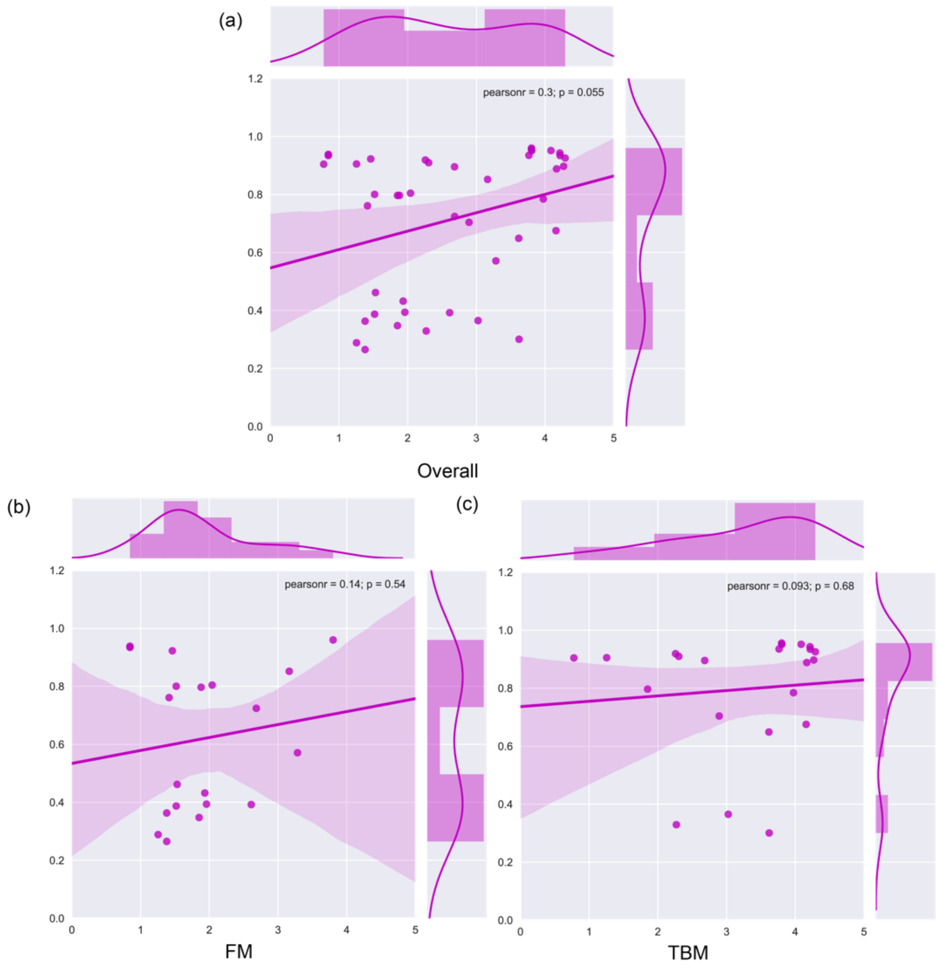

Figure S8. Correlation between model quality and the logarithm of effective alignment depth in MSA for our method tested in the CASP13 set. **(a)** All CASP13 targets. **(b)** CASP13 FM targets. **(c)** CASP13 TBM targets. The vertical axis represents the TM-score, while the horizontal axis represents the log(N_{eff}).

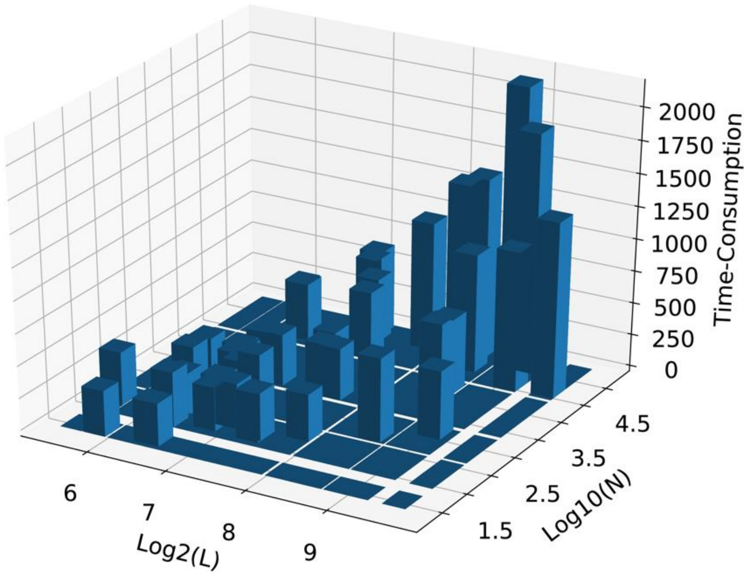

Figure S9. Time consumption of our method on 42 CASP13 targets. *L* represents target length, *N* represents the alignment depth in the MSA and the time consumption is counted by second. Since protein folding via CNS suite is not included and since the model inference is very quick, the computation time is mainly consumed for feature generation.

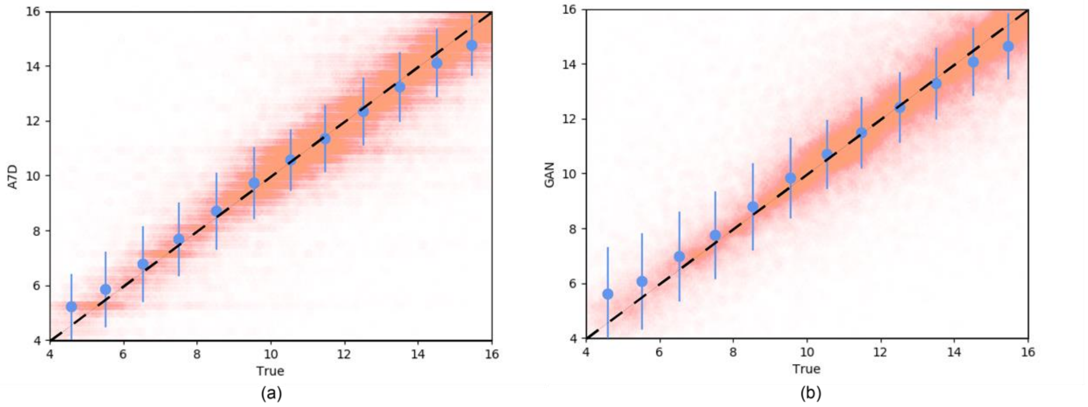

Figure S10. Comparison between distance predictions of A7D and our method. Coordinate scale of both axes are Å. The predicted distances are shown in orange dots. Data marked in blue are mean ± s.d. calculated for 1 Å bins. **(a)** The mode of the distance distributions predicted by A7D plotted against the true distances. **(b)** Similar pairwise comparison between distances predicted by our method and corresponding ground truths.

Table S1. Performance of the ResNet and cGAN models in the validation set.

Predicted distance interval (Å)	ResNet	cGAN
4-6	0.641	0.999
6-8	0.871	1.051
8-10	1.464	1.981
10-12	1.721	2.202
12-14	2.225	2.075
14-16	2.557	2.278
Overall (4-16)	1.832	1.938

Here, we used the mean absolute error (Å) to evaluate the prediction errors for residue pairs with predicted distances falling within various intervals.

Table S2. Model performance for different architectures of G.

	Interval of predicted distance (Å)						
	4-6	6-8	8-10	10-12	12-14	14-16	4-16
ResNet: 2 layers per block	0.973	1.087	1.952	2.136	2.109	2.281	1.920
**ResNet: 3 layers per block (bottleneck channel)**	**0.755**	**0.915**	**1.412**	**1.735**	**1.889**	**2.105**	**1.640**
ResNet: 3 layers per block (barrel-like channel)	0.729	0.952	1.414	1.848	2.061	2.287	1.754
ResNet: Bottleneck	0.891	1.048	1.726	2.218	2.324	2.550	2.011
U-Net	1.660	1.510	1.884	4.047	4.865	5.180	4.334
Dense Net	0.859	1.265	2.184	2.879	3.155	3.455	2.696

Model performance is quantified by the mean absolute error (Å) in the 5-fold cross validation. For fair comparison, all architectures were designed with approximately the same computational consumption (~ 5800MiB FLOPs). The picked architecture is marked in bold.

Table S3. Model performance for different kernel sizes of G.

	Interval of predicted distance (Å)						
	4-6	6-8	8-10	10-12	12-14	14-16	4-16
3×3	0.736	0.859	1.253	1.547	1.672	1.894	1.473
5×5	0.726	0.822	1.109	1.381	1.524	1.760	1.351
**7×7**	**0.717**	**0.816**	**1.034**	**1.274**	**1.426**	**1.667**	**1.271**
3×3 with dilation rate of 2	1.011	0.969	1.425	1.644	1.762	1.999	1.571
Separable convolution kernel with size of 3	0.805	0.912	1.370	1.729	1.844	2.040	1.607

Model performance is quantified by the mean absolute error (Å) in the 5-fold cross validation. The networks compared here are completely the same except the kernels. The picked kernel is marked in bold.

Table S4. Model performance for different activation functions of G.

	Interval of predicted distance (Å)						
	4-6	6-8	8-10	10-12	12-14	14-16	4-16 Å
Leaky-ReLU	0.736	0.859	1.253	1.547	1.672	1.894	1.473
ELU	0.715	0.825	1.181	1.445	1.570	1.817	1.392
C-ReLU	0.815	0.974	1.375	1.757	1.898	2.115	1.656
P-ReLU	0.702	0.808	1.180	1.440	1.574	1.824	1.395
R-ReLU	1.635	1.614	1.676	2.441	2.864	3.164	2.561
tanh	0.862	1.265	2.183	2.876	3.153	3.450	2.693
Softplus	0.690	0.792	1.152	1.391	1.530	1.789	1.361
Softsign	1.045	1.235	1.940	2.529	2.595	2.823	2.264
Swish	0.688	0.792	1.147	1.401	1.521	1.780	1.357
**Swish (with parameter)**	**0.670**	**0.790**	**1.131**	**1.388**	**1.509**	**1.751**	**1.340**

Model performance is quantified by the mean absolute error (Å) in the 5-fold cross validation. The networks compared here are completely the same except the activations. The picked activation is marked in bold.

Table S5. Model performance for different loss functions of G.

	Interval of predicted distance (Å)						
	4-6	6-8	8-10	10-12	12-14	14-16	4-16
**L1 Loss**	**0.736**	**0.859**	**1.253**	**1.547**	**1.672**	**1.894**	**1.473**
L2 Loss	1.482	1.329	2.370	2.548	3.075	5.372	3.766
Huber Loss	1.310	1.307	2.083	2.316	2.318	3.115	2.330
Logcosh loss	1.132	1.060	1.440	1.751	2.035	2.961	1.986
LogMSE loss	1.878	1.657	2.104	1.944	2.467	3.540	2.482
PercentageMAE loss	-	-	-	-	-	-	-

Model performance is quantified by the mean absolute error (Å) in the 5-fold cross validation. The networks compared here are completely the same except the losses. It is noteworthy that for Huber loss, we did a series of experiments at different values of its parameter δ and listed the best performing one here. "-" means training failure. The picked loss function is marked in bold.

Table S6. Model performance for different attention adding strategies of G.

	Interval of predicted distance (Å)						
	4-6	6-8	8-10	10-12	12-14	14-16	4-16
Without attention module	0.736	0.859	1.253	1.547	1.672	1.894	1.473
**Attention module on input only**	**0.704**	**0.787**	**1.139**	**1.425**	**1.547**	**1.749**	**1.351**
Attention module in each block	0.827	0.915	1.388	1.622	1.698	1.841	1.501
Attention module in each layer	1.988	1.859	2.873	4.339	4.622	4.572	3.943

Model performance is quantified by the mean absolute error (Å) in the 5-fold cross validation. The networks compared here are completely the same except the attention adding strategies. The picked strategy is marked in bold.

Table S7. Comparison of the average TM-score for structures constructed using predictions of two same-architecture models trained by different-sourced labels.

	Model 1	Model 2
CASP13 set (42 targets)	0.579	0.619
Membrane protein set (416 targets)	0.500	0.516

Model 1 is trained using crystal structures directly from PDB. Model 2 is trained by structures produced by MD simulations.

Table S8. Comparison of the average TM-score for structures constructed using predictions of our GAN system as constraints against those using DeepConPred2 on CASP12 targets.

	Average TM Score
DeepConPred2	0.409
Our GAN system	0.640

TM-score is averaged over the targets tested.

Table S9. Comparison of the average TM-score for structures constructed using predictions of our GAN system as constraints against the top groups in CASP 13.

	Overall (42 targets)	FM (20 targets)	TBM (22 targets)
QUARK	0.678	0.536	0.816
Zhang-Server	0.676	0.518	0.819
RaptorX-DeepModeller	0.661	0.523	0.786
Our method	0.712	0.620	0.786
	Overall (38 targets)	FM (19 targets)	TBM (19 targets)
A7D	0.702	0.633	0.771
Our method	0.703	0.630	0.775

TM-score is averaged over the targets tested.

Table S10. Comparison of the average TM-score for structures constructed using predictions of our GAN system as constraints against the results of RaptorX server on 38 CAMEO hard targets.

	Average TM Score
RaptorX	0.559
Our GAN system	0.563

TM-score is averaged over the targets tested.
